# Elucidating the Role of Baseline Leukoaraiosis on Forecasting Clinical Outcome of Acute Ischemic Stroke Patients Undergoing Reperfusion Therapy

**DOI:** 10.3390/neurolint14040074

**Published:** 2022-11-10

**Authors:** Stella Karatzetzou, Dimitrios Tsiptsios, Anastasia Sousanidou, Foteini Christidi, Evlampia A. Psatha, Marilena Chatzaki, Sofia Kitmeridou, Erasmia Giannakou, Efstratios Karavasilis, Christos Kokkotis, Nikolaos Aggelousis, Konstantinos Vadikolias

**Affiliations:** 1 Neurology Department, School of Medicine, Democritus University of Thrace, 68100 Alexandroupolis, Greece; 2 Department of Radiology, School of Medicine, Democritus University of Thrace, 68100 Alexandroupolis, Greece; 3 Department of Physical Education and Sport Science, Democritus University of Thrace, 69100 Komotini, Greece; 4 Medical Physics Laboratory, School of Medicine, Democritus University of Thrace, 68100 Alexandroupolis, Greece

**Keywords:** leukoaraiosis, white matter hyperintensities, thrombolysis, thrombectomy, brain revascularization, stroke outcome

## Abstract

Stroke stands as a major cause of death and disability with increasing prevalence. The absence of clinical improvement after either intravenous thrombolysis (IVT) or mechanical thrombectomy (MT) represents a frequent concern in the setting of acute ischemic stroke (AIS). In an attempt to optimize overall stroke management, it is clinically valuable to provide important insight into functional outcomes after reperfusion therapy among patients presenting with AIS. The aim of the present review is to explore the predictive value of leukoaraiosis (LA) in terms of clinical response to revascularization poststroke. A literature research of two databases (MEDLINE and Scopus) was conducted in order to trace all relevant studies published between 1 January 2012 and 1 November 2022 that focused on the potential utility of LA severity regarding reperfusion status and clinical outcome after revascularization. A total of 37 articles have been traced and included in this review. LA burden assessment is indicative of functional outcome post-intervention and may be associated with hemorrhagic events’ incidence among stroke individuals. Nevertheless, LA may not solely guide decision-making about treatment strategy poststroke. Overall, the evaluation of LA upon admission seems to have interesting prognostic potential and may substantially enhance individualized stroke care.

## 1. Introduction

Stroke represents not only the second leading cause of death but also the major cause of acquired disability among adult individuals, mostly accompanied by a considerable unfavorable effect on the long-term functional independence of stroke survivors [1,2,3]. Taking into account the age-related nature of the disease, as almost two-thirds of all stroke patients are aged over 65 [4], in parallel with the ongoing global population growth and the substantial improvement in life expectancy [5], it is anticipated that the overall stroke burden will be significantly enhanced, coupled with a constantly increasing number of stroke survivors. 

The beneficial effects of reperfusion therapy following stroke, either with intravenous thrombolysis (IVT) or mechanical thrombectomy (MT), have been well documented. IVT with recombinant tissue plasminogen activator (rt-PA) has emerged as a first-line treatment strategy for patients with acute ischemic stroke (AIS) within 4.5 h of symptom onset [6]. Increasing evidence suggests the favorable safety and efficacy profile of rt-PA use in a setting of acute stroke management, as prompt IVT implementation has tremendously improved clinical outcomes for stroke individuals presenting within the recommended therapeutic time window [7,8,9]. Regarding the therapeutic approach utilized among patients with AIS, which is specifically attributed to a large vessel occlusion (LVO), MT constitutes the standard of care and has gained international recognition [10,11,12,13,14]. However, the clinical response to the revascularization process appears to remarkably vary between different stroke survivors [15,16]. Indeed, a significant proportion of stroke patients do not achieve a sufficient level of independence poststroke, despite successful recanalization and a good reperfusion status accomplished, reflecting the challenging nature of AIS treatment. 

Given the fact that prompt forecasting of each patient’s propensity for recovery substantially contributes to the decision-making in terms of poststroke treatment strategy, it becomes essential to be provided with an accurate and timely outcome prognosis [17]. Several variables have been identified and further explored in terms of prognostic potential poststroke, including several blood biomarkers [18], neurophysiological techniques [19], and even artificial intelligence approaches [20]. Understanding the factors mediating futile recanalization and, thus, adversely influencing functional outcomes following stroke, is of great interest in identifying patients at high risk of unfavorable prognosis and, therefore, appears to be paramount to individualizing patients’ selection for both IVT and MT and optimizing overall stroke care. Up to today, the role of several clinical and imaging variables has been investigated in an acute stroke setting regarding their prognostic value after IVT or MT is performed, leukoaraiosis being among them. 

Introduced by Hanchski in 1987 [21], the term leukoaraiosis (LA) stands for a radiological phenomenon that refers to brain white matter abnormalities of ischemic origin and may be described as either focal hypointensities on computed tomography (CT) scans or white matter hyperintensities (WMHs) on T2-weighted or fluid-attenuated inversion recovery (FLAIR) magnetic resonance images [22,23]. Baseline LA evaluation, being indicative of brain reserve and collateral flow efficiency, mirrors the intrinsic susceptibility of brain tissue to acute ischemic insults and appears able to provide important insight into the brain capacity for adaptation and reorganization following stroke [24]. As a result, a greater LA burden is mostly accompanied by a lower tolerance of brain parenchyma to emerging ischemia, leading to earlier and irreversible infarcts. Representing an imaging sign of neurodegeneration, LA is commonly encountered among the elderly, with advanced age being coupled with significantly increasing prevalence of white matter lesions [25,26]. 

Considering the expected substantial increase in stroke patients with pre-existing LA undergoing reperfusion therapy, as stroke burden rises among the elderly and life expectancy continues to significantly expand in developed countries, it is of key importance to elucidate the potential contribution of LA on forecasting clinical response to IVT or MT poststroke. Thus, the objective of our study was to review all available literature published within the last decade dealing with baseline LA as a prognostic tool following stroke recanalization.

## 2. Materials and Methods

The Preferred Reporting Items for Systematic Reviews and Meta-Analyses (PRISMA registration number: CRD42022369840) were used to guide this study. Our study’s methods were a priori designed. 

### 2.1. Search Strategy

Two investigators conducted literature research on two databases (MEDLINE and Scopus) (SK and AS) to trace all relevant studies published between 1 January 2012, and 1 November 2022. Search terms were as follows: (“leukoaraiosis” OR “white matter hyperintensities” OR “WMHs”) AND (“thrombolysis” OR “thrombectomy” OR “brain revascularization” OR “reperfusion”). The retrieved articles were also hand-searched for any further potential eligible articles. Any disagreement regarding the screening, or selection process, was solved by a third investigator (KV) until a consensus was reached. 

### 2.2. Selection Criteria

Only full-text original articles published in the English language were included. Secondary analyses, reviews, guidelines, meeting summaries, comments, unpublished abstracts, or studies conducted in animals were excluded. There was no restriction on study design or sample characteristics.

### 2.3. Data Extraction

Data extraction was performed using a predefined data form created in Excel. We recorded the authors, year of publication, number of participants, follow-up time, method of leukoaraiosis assessment, time of computed tomography execution, time from symptom onset to recanalization, the scale used to examine stroke severity and clinical outcomes, and the main results of each study.

### 2.4. Data Analysis

No statistical analysis or meta-analysis was performed due to the high heterogeneity among studies. Thus, the data were only descriptively analyzed. 

## 3. Results

### 3.1. Database Searches

**Figure 1:**
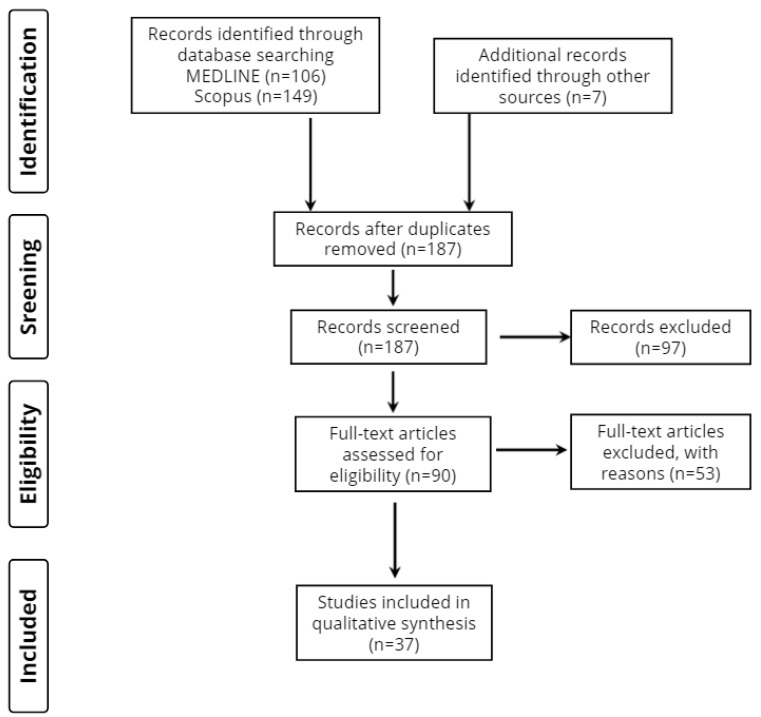
Study flow diagram (PRISMA flowchart).

Overall, 262 records were retrieved from the database search. Duplicates and irrelevant studies were excluded; hence, a total of 90 articles were selected. After screening the full text of the articles, 37 studies were eligible for inclusion (Figure 1).

### 3.2. Study Characteristics

A total of 37 publications fulfilled our inclusion criteria, as shown in Table 1. Sixteen focused on endovascular stroke therapy, 15 of which studied mechanical thrombectomy and one intra-arterial thrombolysis, 18 examined intravenous thrombolysis, and 3 studied patients who underwent intravenous thrombolysis and/or endovascular stroke therapy. Considering the origin of the studies, 14 were from Asia, 14 were from Europe, 7 were from America, and 2 were from Australia.

### 3.3. Method of LA Neuroimaging Assessment

In total, 20 studies utilized the Fazekas score, 11 the van Swieten scale, three the Age-Related White Matter Change Scale, two differentiated between the presence or absence of LA, one used the CREDOS WMH visual rating scale, and four estimated WMH volume on imaging. 

### 3.4. Study Design

In total, all the studies included in this review were longitudinal. They were either retrospective or prospective cohorts.

### 3.5. Stroke Patient Groups and Demographic Profile

The total number of stroke patients included in all studies ranges from n = 56 [31] to n = 3017 [46]. Across the 37 studies, 6 studies have a disease sample size between 1–100 patients, 12 studies between 101–200, 5 studies between 201–300, 2 studies between 301–400, and 12 studies have a disease sample size larger than 400 patients. The mean/median patients’ age ranges from 64.4 [37] years to 81.3 [47] years.

### 3.6. Reference Groups

In none of the 37 included studies, stroke patients are contrasted to demographically-matched healthy individuals and none of the studies include a disease-control group other than stroke patients.

### 3.7. Scales of Stroke Severity and Prognosis/Clinical Outcome

National Institutes of Health Stroke Scale (NIHSS) and modified Rankin Scale (mRS) have been simultaneously used in 30 studies. NIHSS was the only scale in three studies and mRS exclusively in one study. In two studies NIHSS was combined with the Fugl-Meyer rating scale (FMS) and in one study with the Oxford Handicap Scale (OHS).

## 4. Discussion

A literature review over the last decade was conducted in order to elucidate baseline LA prognostic value in AIS patients undergoing reperfusion therapy. A total of 37 full-text original articles dealing with the potential utility of LA evaluation in forecasting stroke survivors’ clinical response to revascularization were identified and classified into three groups based on the implemented revascularization technique.

### 4.1. Endovascular Stroke Therapy

With respect to poststroke outcome after EST, Zhang et al. [28], having studied 129 AIS patients treated with MT, found an inverse correlation between pre-existing LA and 3-month functional outcome after MT, as severe LA identified on initial head CT was independently associated with an unfavorable outcome in patients undergoing MT. Thus, stroke individuals with baseline LA of high severity were found to be at increased risk for poor recovery after MT compared to patients with absent or only mild LA. Similarly, Guo et al. [32], in an attempt to better clarify factors potentially able to impact outcome after MT, enrolled 251 stroke patients and reported LA severity as an independent predictor for both futile recanalization (FR) and mortality at 3 months poststroke. Unlike patients with absent-to-moderate LA burden, stroke survivors with severe LA were accompanied by a rather diminished chance of a favorable clinical outcome after MT, thus establishing LA severity as a significant MT efficiency indicator. In line with the aforementioned studies’ results, Mutzenbach et al. [38], having examined 209 patients with AIS treated with MT, showed that approximately only one-fifth of individuals with severe LA achieve a good functional status at 3 months poststroke despite successful revascularization, thus highlighting the highly predictive value of pre-existing LA regarding MT clinical outcome and the role of severe LA as an independent risk factor for poor recovery after MT. Interestingly, the degree of white matter abnormalities was demonstrated to be positively associated with age, a linear relationship that appears to be of key importance within an aging population with higher odds of stroke occurrence and MT implementation.

Additionally, Liu et al. [34] further explored the potential linkage between LA severity and functional outcome after complete reperfusion by MT in a population of 97 stroke patients and concluded that greater baseline LA burden seems to be indicative of unfavorable outcome after MT in patients with AIS attributed to LVO. More specifically, the risk of either long-term disability or death among stroke patients with moderate-to-severe LA was found to be more than three times higher when compared to stroke survivors presented with absent-to-mild LA, thus implying that initial LA may exhibit useful prognostic potential in cases of AIS patients undergoing MT. Furthermore, Benson et al. [40] investigated the potential relationship between the degree of LA present on baseline head CT and clinical outcome after endovascular treatment in 174 stroke patients. They observed that the severity of white matter lesions was significantly correlated with recovery propensity after MT, with a higher degree of LA being coupled with a worse functional outcome at 3 months, even among patients with successful recanalization. The researchers came to the conclusion that LA assessment may serve as an independent prognostic marker after MT, thus enhancing the predictive ability of the initial CT scan in an acute stroke setting.

As far as the role of brain atrophy and LA in forecasting futile recanalization poststroke is concerned, Kaginele et al. [41], having evaluated a total of 175 patients admitted for MT with complete reperfusion, showed that the extent of pre-existing white matter abnormalities visually graded on admission head CT scan is significantly associated with futile recanalization, as the group of stroke patients with higher brain atrophy/LA severity was characterized by a poor clinical response to MT. It is noteworthy that the researchers reported brain atrophy/LA degree as a powerful surrogate marker of pre-stroke functional status including brain biological age and comorbidities, capable of reliably predicting MT treatment effect and differentiating patients at increased risk for futile recanalization from those with an expected favorable outcome. Thus, determination of baseline brain atrophy/LA in stroke patients seems to be of great clinical value, in terms of appropriate patient selection for efficacious MT utilization and individualization of decision-making. Apart from that, Pedraza et al. [35], in an attempt to explore the role brain atrophy is to play in determining the risk of futile revascularization among AIS patients treated with MT, studied a population of 295 stroke individuals. They observed that the severity of brain atrophy was inversely correlated with clinical outcomes after MT, exerting a substantial impact on the response to endovascular treatment. Interestingly, the influence of pre-existing brain atrophy on the level of functional independence achieved after MT was found to be independently amplified by each patient’s chronological age and acute cerebral infarct volume. More specifically, it was shown that stroke patients with advanced age and greater infarct volume with present brain atrophy on admission CT scan were more likely to exhibit insufficient clinical recovery after MT and be left disabled or even dead, despite prompt and complete reperfusion.

With regard to clinical and radiographic variables able to act as predictors of futile recanalization among AIS patients undergoing MT, Gilberti et al. [30], having enrolled 68 patients, assessed the potential link between the extent of LA and functional outcome after MT, as well as the role of baseline NIHSS score, age, and time to treatment. According to the study’s results, both LA severity and NIHSS score on admission were identified as independent predictors of poor clinical response to MT, whereas age and longer delay to reperfusion therapy were not found to be significantly associated with functional recovery after MT, suggesting the valuable prognostic potential of LA evaluation in conjunction with initial NIHSS score determination. Moreover, Guo et al. [37], in an attempt to provide important insight into the impact of LA burden on earlier neurological outcomes in acute stroke patients admitted for MT, examined a population of 273 patients and reported a negative correlation between the degree of pre-existing LA and early neurological improvement, as the group of patients characterized by severe baseline LA was accompanied by significantly lower rates of early neurological improvement when compared to those individuals with absent-to-moderate LA. When excluding patients with symptomatic intracranial hemorrhage (ICH), severe LA was shown to be well-correlated with increased odds of early neurological deterioration, thus pointing out its potential role as an independent predictor of early neurological deterioration after MT in patients with AIS originated from LVO.

Regarding the relationship between both LA severity and time to successful reperfusion with 3-month functional outcome in an acute stroke setting, Milkati et al. [36] explored the influence of LA presence and extent on admission head CT scan not only on clinical response to MT but also on the association between onset-to-reperfusion time and 3-month functional outcome. The researchers concluded that LA burden was independently related to a poor outcome, despite successful reperfusion after MT, while inversely affecting onset-to-reperfusion time needed to achieve similar outcomes. In order for complete revascularization to be coupled with a favorable clinical outcome, it had to take place substantially earlier in stroke patients with initially identified high LA burden than in patients with absent or only mild LA.

With respect to the occurrence of hemorrhagic transformation (HT), Shi et al. [27] investigated the potential role of LA severity as a predictor of symptomatic HT and subsequently unfavorable outcomes in AIS patients treated with MT. Moderate-to-severe LA was demonstrated to parallel with increased rates of HT, as well as independently forecasting the occurrence of brain parenchymal hematoma (PH) in stroke patients after endovascular treatment. The latter is of clinical relevance because PH-complicating reperfusion is often accompanied by worse functional recovery.

Apart from that, Lee et al. [42] reported that baseline cerebral microbleeds (CMBs) burden is considered able to influence clinical outcomes after MT, with an increase in detectable CMBs being coupled with a substantially poorer functional recovery among stroke patients undergoing MT. Interestingly, the negative impact of CMBs on MT outcomes was mainly mediated by a greater small vessel disease burden as reflected in the severity of WMHs, followed by an increased rate of post-procedural hemorrhagic complications, including HT. Another factor that may hinder functional recovery in stroke patients presented with a degree of CMBs is the incidence of utile recanalization with lower rates of successful reperfusion being accompanied by worsened clinical outcomes post-MT.

On the contrary, Atchaneeyasakul et al. [31], having studied a total of 56 stroke patients receiving mechanical thrombectomy with stentriever devices, reported no significant association between white matter lesions extent and occurrence of HT and PH, as LA severity was not found to influence the odds of hemorrhagic complications or death after MT. Similarly, Boulouis et al. [33], in an attempt to provide further insight into the relationship between WMH burden and functional outcome after MT among stroke subjects, observed that although WMH severity appeared to be independently associated with an unfavorable MT prognosis poststroke, an increased volume of white matter abnormalities was not significantly associated with higher rates of either sICH or reperfusion success, thus highlighting the safety and efficacy profile of MT in terms of sICH occurrence and recanalization achievement, respectively. It is of great interest, that more than 1 out of 4 patients presented with high WMH burden achieved a good functional outcome post-ΜΤ, suggesting that increased baseline WMH volume may not solely account for MT exclusion criterion. Additionally, Mechtouff et al. [39] evaluated the impact of the degree of pre-existing LA on the extent of functional recovery among 293 AIS patients after MT and showed that the presence of white matter abnormalities does not seem to affect the clinical outcome at 3 months after endovascular therapy, as well as the rate of futile recanalization.

As for the prognostic utility of LA burden in patients presented with AIS due to large cerebral artery occlusion undergoing intra-arterial therapy (IAT), Giurgiutiu et al. [29], having enrolled 95 patients, investigated the potential association between pre-existing LA severity and clinical response in a setting of IAT. Although greater LA volume appeared to be linked with poor collateral flow efficiency, LA severity was not found to be significantly indicative of long-term functional outcome, in contrast to intravenous tissue plasminogen activator use and recanalization status, which independently predicted a favorable outcome after IAT.

### 4.2. Intravenous Thrombolysis

With respect to the clinical response to IV rt-PA utilization in ischemic stroke patients in the setting of detectable LA on baseline CT scan, Huang et al. [43] examined 101 AIS patients receiving IVT to elucidate factors able to influence prognosis following IV tPA administration. When compared to stroke survivors without LA, patients with pre-existing LA were more likely to have an unfavorable prognosis following IVT, with white matter changes severity being independently associated with the therapeutic effect of IV tPA use. Moreover, Liu et al. [51], to provide further insight into the impact different LA degrees exert on functional outcomes among patients with AIS treated with IVT, studied 97 stroke individuals within the recommended time window of 4.5 h after symptom onset and concluded that clinical recovery and prognosis following IVT depended on the initial LA severity, with different LA burden being accompanied by different level of independence at 3-months follow-up. Interestingly, the presence of mild LA on baseline CT scan was well correlated with early neurological recovery and a favorable motor functional outcome compared to absent or moderate-to-severe degree of LA, whereas patients presented with moderate-to-severe LA were more prone to develop both HT and stroke recurrence. During the 3-month follow-up, moderate-to-severe LA was reported to act as an independent prognostic marker of poor functional outcome.

Additionally, Zhong et al. [49], having enrolled 79 ischemic stroke patients undergoing IVT, explored the potential linkage between pre-existing LA burden and the achieved level of reperfusion after IVT. The researchers demonstrated that severe LA on baseline head CT was significantly associated with both infarct expansion and unfavorable clinical outcome, independently of the reperfusion status. Thus, LA severity evaluation on admission may not play a substantial role in forecasting reperfusion insufficiency. Apart from that, the effect of white matter lesions’ location on the therapeutic effect and clinical prognosis following IVT was investigated by Liu et al. [54] within a population of 113 ischemic stroke patients treated with IV tPA use. Classified into deep white matter hyperintensity (DWMH) and periventricular hyperintensity (PVH), white matter abnormalities detected at different brain sites were found to differentially affect the efficacy of IVT. More specifically, the presence of DWMH, reflecting chronic hypoperfusion, was significantly linked to increased incidence of both early neurological deterioration and HT after IVT compared to PVH. Unlike DWMH, PVH detectable on baseline CT scan was reported as an independent risk factor of stroke recurrence, as well as enhanced the risk of poor motor functional outcome. However, there was no significant difference between patients with DWMH and those with PVH in terms of motor recovery at 3-months follow-up.

As far as the role both early ischemic and pre-existing brain imaging signs are to play in terms of predicting clinical response to IVT in ischemic stroke patients, Delcourt et al. [56] observed that radiographic markers of “brain frailty”, including LA and brain atrophy, were reliably predictive of both poor clinical outcome and death, as well as were significantly correlated with a diminished chance of independence during a 3-month follow up. The presence of brain atrophy was, nevertheless, accompanied by a decreased rate of early mortality. Furthermore, a secondary analysis of the IST-3 trial [46] evaluated the relationship between brain imaging markers and functional recovery in AIS patients receiving IV tPA. As assessed on baseline head CT scan, LA and severe atrophy were not only indicative of death at 6 months poststroke but also were coupled with a significantly reduced chance of a long-term favorable outcome. However, any extent of LA was not found to be associated with the development of symptomatic ICH. It is of interest that there was no interaction detected between any individual or combined brain imaging signs and effects of IVT on the reported outcomes, either functional recovery or symptomatic ICH occurrence.

Considering that the assessment of the global burden of small vessel disease (SVD), as reflected by both white matter changes and pre-existing lacunar infarcts, may constitute a more precise marker with valuable prognostic potential in terms of clinical outcome and hemorrhagic complications following IVT among stroke patients, Arba et al. [48] investigated the association between detectable on baseline CT LA and lacunes and subsequent response to IV tPA. The researchers concluded that the presence and severity of SVD burden were predictive of poor clinical outcomes and significantly linked to symptomatic HT after IVT. Apart from that, the absence of LA, among other variables, was shown to be an independent predictor of favorable outcomes at 3 months poststroke in 394 patients treated with IVT in a study conducted by Zivanovic et al. [57].

Considering the influence of pre-existing LA on CT scans of stroke patients admitted for IVT on 3-month functional outcome and risk of symptomatic ICH, Yang et al. [53] examined a population of 612 stroke individuals to evaluate the correlation between baseline LA burden and prognosis after IVT. Severe LA was shown to be independently predictive of poor clinical recovery at 3 months poststroke, while a great LA degree was not found to be accompanied by a higher occurrence rate of any post-tPA hemorrhage or symptomatic ICH following IVT. The researchers concluded that, although exhibiting valuable prognostic potential, LA severity should not solely determine the decision-making regarding stroke therapeutic strategy. On the contrary, Qiu et al. [59], having enrolled 175 ischemic stroke patients eligible for IVT, reported an independent association between white matter hypoperfusion related to LA and ICH following IV tPA infusion, thus highlighting the role of admission LA as a marker of post-IVT ICH development. However, a similar correlation was not observed between LA burden and outcome prognosis, with no significant difference in terms of the clinical outcome being detected in hypoperfusion compared to the normal perfusion group.

Despite being associated with ICH occurrence and 3-month unfavorable outcome following IVT, LA severity was not reported as an independent prognostic marker of symptomatic ICH, mortality, or clinical outcome in a study conducted by Capuana et al. [60] within a population of 434 ischemic stroke patients treated with IVT. The researchers pointed out that identified moderate-to-severe LA on baseline CT scans of stroke individuals should not stand for a contraindication of IV tPA use, as those patients may still benefit from reperfusion therapy, despite the increased risk of symptomatic ICH being described. Apart from that, Mcalpine et al. [44], having evaluated a total of 158 AIS patients investigated the potential linkage between initial LA severity and early neurological recovery following IVT, which is commonly well correlated with a favorable prognosis and decreased mortality poststroke. In the setting of AIS, LA identification on admission CT scan was found to have no impact on short-term clinical outcome, thus being unable to reliably predict the likelihood of early neurological improvement after IVT.

With respect to the prognostic significance of LA in terms of tPA-related bleeding events, Willer et al. [45], having enrolled 311 stroke individuals undergoing IVT, investigated the contribution of brain atrophy and/or LA to the risk of either symptomatic or asymptomatic ICH following IV tPA utilization. The presence of LA on the initial CT scan was found to be accompanied by a two-fold risk for HT after IVT in the study population, although not standing as an independent predictor of post-IVT hemorrhage. Contradictory results were reported in a study conducted by Nagaraja et al. [52], having examined 366 ischemic stroke patients admitted for IVT, observed a significant association between LA severity and poor clinical outcome, but not with the development of HT or sICH after IVT.

Regarding the occurrence of remote post-thrombolytic hemorrhagic complications of IVT, Chen et al. [55] explored the frequency and the potential correlation between ICH out of the initial ischemic lesion and the extent of white matter abnormalities in patients presenting with acute ischemic cerebral infarction receiving IVT. The researchers demonstrated a positive correlation between the severity of white matter changes and the occurrence rate of remote ICH, as a more extensive white matter hyperintensity profile was independently coupled with increased risk of remote ICH and subsequent unfavorable functional outcome. Additionally, Prats-Sanchez et al. [50], in an attempt to differentiate between risk factors for deep and lobar remote hemorrhages after IVT, assessed 934 ischemic stroke patients treated with IV tPA use and concluded that the development of different sites’ parenchymal hematomas following IVT were mediated by different mechanisms. More specifically, deep remote brain hemorrhages were attributed to hypertensive episodes shortly after tPA administration, while the occurrence of lobar remote bleeding events was associated with imaging markers of cerebral amyloid angiopathy, LA being among others.

As far as the prognostic potential of LA burden among elderly stroke patients is concerned, Nighoghossian et al. [47], having studied 180 stroke survivors aged over 75 years, evaluated the influence of LA severity on hemorrhagic risk within this subgroup after IVT. Severe LA was shown to independently enhance the prediction of post-thrombolytic parenchymal hematomas’ development, which was accompanied by remarkable clinical deterioration. Compared to infarct size, LA burden was found to perform better in terms of forecasting sICH. Similarly, Zhang et al. [58], in an attempt to provide important insight into the relationship between pre-existing LA extent, HT, and 3-month prognosis among stroke patients aged ≥ 60 years, examined a total of 125 elderly stroke individuals and observed a positive correlation between LA severity and bleeding risk. More specifically, patients with high LA burden were more prone to both HT and poor prognosis after IVT, with no detectable risk increase regarding symptomatic ICH occurrence and fatal hemorrhage. Finally, Sudre et al. [63] enrolled a population of 357 stroke patients older than 80 having undergone IVT and/or EST and reported a significant association between a favorable outcome at 3 months and absence of high severity LA on admission, as described by Fazekas 3 grade periventricular WMHs.

### 4.3. Intravenous Thrombolysis and/or Endovascular Stroke Therapy

Regarding the prognostic potential of endothelial dysfunction markers as the soluble tumor necrosis factor-like inducer of apoptosis (sTWEAK) in an acute ischemic stroke setting, da Silva-Candal et al. [61] explored the potential linkage between sTWEAK, LA, and HT and intended to elucidate their role in forecasting functional outcome among stroke patients undergoing reperfusion therapies. The researchers studied a population of 875 stroke individuals treated with IVT, MT, or both and found a strong correlation between admission serum levels of sTWEAK and clinical outcome after reperfusion treatment, with a reported 13-fold higher risk of unfavorable 3-month prognosis in stroke patients presented with increased sTWEAK plasma concentration. It is noteworthy that the effect of sTWEAK levels on outcome after recanalization procedures may be mediated by the presence and severity of baseline LA and its association with HT development. Similarly, Hervela et al. [62] investigated the relationship between LA burden, sTWEAK baseline levels, and stroke recurrence in order to yield additive information regarding LA impact on stroke management and concluded that both LA severity and sTWEAK concentrations at admission were strong predictors of stroke recurrence in ischemic stroke subjects having undergone IVT, MT or IAT. Interestingly, LA and sTWEAK appeared to be interrelated variables, thus suggesting that sTWEAK may act as a surrogate marker for LA, as well as a therapeutic target poststroke.

## 5. Conclusions

Taking everything into consideration, the present review provides evidence regarding the prognostic significance of LA severity, as evaluated on baseline head imaging within the early phase following stroke. Our findings suggest the significant role LA assessment may play in forecasting clinical response to reperfusion treatment strategy. LA burden, serving as a surrogate marker of biological age and consequently “brain frailty” among stroke patients, appears to be able to yield additional information in terms of cerebral reserve and brain parenchyma resilience to emerging ischemia, thus reliably predicting both reperfusion status and clinical outcome after either IVT or MT. Determining the extent of pre-existing white matter abnormalities may properly guide the decision-making in a setting of acute stroke, as a greater degree of these lesions is usually coupled with unfavorable prognosis and poorer outcomes after the selected intervention is implemented. Additional data support the prognostic value of baseline LA on the potential development of adverse hemorrhagic events after IVT or MT, with a higher LA burden identified on admission head imaging being accompanied by an increased incidence of intervention-related ICH. Nevertheless, LA assessment should be interpreted in a clinical context and may constitute a more powerful tool when being paralleled with other clinical and neuroimaging biomarkers in an attempt to facilitate individualized stroke care. The aforementioned results highlight the need to further investigate LA as a promising imaging marker for clinical outcomes after IVT or MT to enhance patients’ risk stratification and promote overall stroke management. Additional studies among stroke individuals on the linkage between LA burden and prognosis after stroke intervention are recommended in order to provide further insight into this clinically important relationship.

## Figures and Tables

**Table 1:** Characteristics of the 37 included studies.

	Authors, Year of Publication	Number of Participants/Age of Participants	Follow-Up Time	LA/WMH Assessment	Time of Neuroimaging	Time to Recanalization	Scale of Stroke Severity and Prognosis/Clinical Outcome	Main Results
Endovascular stroke therapy								
1.	Shi et al., 2012 [27]	105/ 65.9 ± 18.9	Until discharge	Fazekas scale	On admission and 3–12 h after MT	median of 5–5.8 h from symptom onset	NIHSS on admission, mRS at discharge	Patients with moderate or severe LA had worse clinical outcomes at discharge and higher rates of in-hospital mortality. Also, severity of LA was an independent predictor of hemorrhagic transformation and parenchymal hematoma
2.	Zhang et al., 2014 [28]	129/ 71 (58–80)	90 days	Van Swieten scale	On admission and within 7 days	NA	NIHSS on admission, mRS scores at the time of presentation, discharge, and at 90 days	Severe LA was independently associated with a poor outcome at 90 days after endovascular stroke therapy. Moreover, among patients who were alive at discharge, those with severe LA had significantly less frequent improvement in the mRS score from discharge to 90 days
3.	Giurgiutiu et al., 2015 [29]	73/ 67.2 (SD 15.7)	90 days	WMH Volumetry	Pre-intervention and within 24 h	within 6 h from symptom onset	NIHSS on admission, mRS at day 90	Good collateral circulation grade in AIS patients undergoing IAT was independently associated with reduced LA volume, and higher LA volume decreased the odds of a good outcome, but LA did not appear to be a contraindication for acute intervention
4.	Gilberti et al., 2017 [30]	68/ 74 (IQR 66–79)	90 days	van Swieten scale	On admission and within 24 h after MT	mean of 245.1 min from symptom onset	NIHSS at baseline, mRS at day 90	The presence of moderate to severe LA was an independent predictor of futile recanalization
5.	Atchaneeyasakul et al., 2017 [31]	56/ 67.3 ± 14.2	90 days	WMH Volumetry	On admission and within 24 h after MT	within 6 h from symptom onset	mRS at discharge and at 90 days	Increasing WMH volume did not significantly affect the odds of 90-day good outcome, intracerebral hemorrhage, parenchymal hematoma, successful recanalization, or death after mechanical thrombectomy using stentrievers
6.	Guo et al., 2019 [32]	251/ 64.4 (SD 11.8)	90 days	Van Swieten scale	Pre-intervention	mean of 361 min from symptom onset	NIHSS pre-treatment, mRS pre-treatment and at 90 days	Severe LA was a significant predictor of poor functional outcome at 90 days in acute stroke patients undergoing mechanical thrombectomy with stent-retriever devices. Additionally, the prevalence of futile recanalization and the mortality rate in patients with severe LA was higher than that in patients with absent-to-moderate LA, but the rate of symptomatic ICH was similar between the two groups
7.	Boulouis et al., 2019 [33]	496/68.1 ± 15.0	90 days	WMH Volumetry	Baseline MRI	within 5 h of symptom onset	NIHSS at baseline, mRS at 90 days	Patients exhibited increasingly worse outcomes with increasing WMH severity, but WMH volume was not associated with sICH rate, nor did it influence recanalization success. More than a quarter of patients in the highest WMH quartile experienced favorable 3-month outcomes, which suggests that MT should not be denied in patients with a high WMH burden
8.	Liu et al., 2019 [34]	97/ 70.0 ± 12.4	90 days	Fazekas scale	On admission and within 24 h after MT	mean of 338 min from symptom onset	NIHSS on admission, mRS at 3 months	Patients with moderate to severe LA had more than 3 times higher risk of having poor 90-day outcomes compared to patients with absent to mild LA, after successful recanalization. Also, patients with higher LA scores appeared to have an increased risk of any type of hemorrhage or symptomatic ICH, but the difference did not reach statistical significance
9.	Pedraza et al., 2020 [35]	295/ 71.29 ± 13.27	3 months	Fazekas scale	On admission	NA	NIHSS at baseline, mRS at baseline and at 3 months	Futile reperfusion (mRS score > 2 at 3 months) was associated with higher scores on the Fazekas scale
10.	Mikati et al., 2020 [36]	144/ 68 (57–81)	90 days	van Swieten scale	On admission	within 24 h from symptom onset	NIHSS at the time of presentation, mRS at admission and at 90 days	Greater pre-existing LA was associated with poor 90-day functional outcome after successful reperfusion and LA impacted the association between the symptom onset-to-reperfusion time and 90-day mRS. Specifically, reperfusion had to occur at least 2 h earlier in subjects with moderate-to-severe LA than in those with absent-to-mild for similar 90-day functional outcomes
11.	Guo et al., 2020 [37]	273/ 64.4 (SD 11.9)	90 days	van Swieten scale	On admission and within 24 h after MT	median of 360 min from symptom onset	NIHSS at baseline and after 24 h, mRS at 90 days	There was a significantly lower ENI rate (defined as a decrease of ≥4 points on the NIHSS, or an NIHSS score of zero 24 h after baseline assessment) and non-significantly higher END rate (defined as an increase of ≥4 points on the NIHSS 24 h after baseline) in patients with severe LA compared with patients with absent-to-moderate LA. However, when the analysis was restricted to patients without ICH, severe LA was a significant independent predictor of END
12.	Mutzenbach et al., 2020 [38]	209/ 75.0 (IQR 63.0–81.0)	3 months	ARWMC scale	NA	mean of 247 min from symptom onset	NIHSS on admission, mRS at 3 months	Severe LA was associated with poor clinical outcomes at 3 months in acute stroke patients undergoing MT due to emergent M1 middle cerebral artery occlusion. Moreover, there were more hospital deaths in the severe LA group than in the absent to moderate LA group, but there were no associations between LA and the presence of ICH
13.	Mechtouf et al., 2020 [39]	293/ 67.12 (SD 16.23)	90 days	Fazekas scale	On admission	mean of 233 min from symptom onset	NIHSS at baseline and at discharge, mRS at day 90	Although WMH severity was moderately associated with a poor outcome, it was not an independent predictor in multivariate analysis. Also, WMHs severity did not influence the risk of parenchymal hemorrhage or the rate of futile recanalization
14.	Benson et al., 2021 [40]	174/ 68.0 ± 9.1	90 days	Fazekas scale	Pre-intervention	NA	NIHSS at baseline, mRS at day 90	LA severity was associated with worse 90-day outcome, even after successful recanalization, and it was an independent risk factor for worse outcomes
15.	Kaginele et al., 2021 [41]	175/ 77.15 (SD 7.09)	90 days	Fazekas scale	Pre-intervention	median of 300 min from symptom onset	NIHSS on admission, mRS at day 90	Increasing brain atrophy and LA severity correlated with decreasing rates of 90-day good functional outcome, suggesting that a simplified, visual assessment of their degree on plain head CT was associated with futile recanalization in patients age > 65 years
16.	Lee et al., 2022 [42]	577/67 ± 13	3 months	CREDOS WMH visual rating scale	CT at baseline, MRI pretreatment	within 24 h of symptom onset	NIHSS at baseline, mRS at 3 months	Increased WMH burden was significantly associated with poorer functional outcomes
**Intravenous Thrombolysis**								
1.	Huang et al., 2013 [43]	101	90 days	Presence or absence of LA	On admission and 24 h after IVT and at clinical deterioration	within 4.5 h from symptom onset	NIHSS at baseline, mRS at day 90	The good outcome group had fewer patients with LA and the absence of LA before thrombolysis was significantly associated with better functional outcome
2.	McAlpine et al., 2014 [44]	158/ 77 (IQR 68-84)	3 months	Van Swieten scale	On admission	within 4.5 h from symptom onset	NIHSS at onset, and at 24 h after IVT, mRS at onset and at 3 months	There was no evidence of the association between the degree of LA and early neurological recovery after IVT
3.	Willer et al., 2015 [45]	311	36 h	Fazekas scale, ARWMC scale	On admission	within 4.5 h from symptom onset	NIHSS	LA doubles the risk of post-thrombolytic hemorrhagic transformation and symptomatic hemorrhage, but it was not an independent predictor of HT
4.	The IST-3 collaborative group, 2015 [46]	3017	6 months	Fazekas scale, van Swieten scale	Pre-intervention and within 24–48 h poststroke and at clinical deterioration within 7 days	within 6 h of symptom onset	NIHSS at baseline, OHS at 6 months	LA and severe atrophy predicted death, reduced chance of being independent, and diminished chance of a favorable outcome at 6 months
5.	Nighoghossian et al., 2016 [47]	180/ 81.3 (SD 4.6)	3 months	Fazekas scale	On admission	mean time of 164.1 min from symptom onset	NIHSS at baseline, mRS at 3 months	In elderly patients treated with intravenous thrombolysis, severe LA was associated with HT and after adjusting for NIHSS and infarct volume it remained the only independent predictor of parenchymal hemorrhage
6.	Arba et al., 2016 [48]	820/ 71.3 ± 13.2	90 days	Van Swieten scale	On admission	within 3 h from symptom onset	NIHSS at baseline, mRS at day 90	In patients younger than 80 years of age, severe SVD (comprising the presence and severity of LA and lacunes on baseline computer tomography scan) consistently reduced the chances to have either excellent or good neurological outcomes. The global burden of SVD was significantly associated with symptomatic hemorrhagic transformation in the whole cohort study, with a fivefold increase of risk in patients with severe SVD
7.	Zhong et al., 2016 [49]	79/ 69.99 ± 11.76	3 months	Fazekas scale	On admission and at 24 h after IVT	within 6 h of symptom onset	NIHSS at baseline, mRS at 3 months	Severe LA was associated with infarct growth and it was also an independent predictor of poor 3 months functional outcome after adjusting for reperfusion and baseline severity of stroke, but the burden of LA did not correlate with reperfusion inefficiency after IVT
8.	Prats-Sanchez et al., 2017 [50]	934/ 73.9 ± 12.6	14 days	Fazekas scale	On admission and within 36 h after IVT	mean of 144 min	NIHSS on admission	Lobar, but not deep, remote parenchymal hemorrhage were associated with the presence of severe LA
9.	Liu et al., 2018 [51]	97/ 66.6 ± 9.1	90 days	Fazekas scale	NA	within 4.5 h from symptom onset	Baseline NIHSS, FMS at baseline and at 90 days	Moderate to severe LA was an independent predictor of 90-day poor functional outcome and the patients in this group had a higher rate of hemorrhagic transformation and recurrent stroke. Interestingly, the percentage of the mild LA group was higher in early neurological recovery than that of the no LA or the moderate to severe LA group
10.	Nagaraja et al., 2018 [52]	366/ 67 ± 15	36 h	Fazekas scale	On admission and at 24 h	NA	NIHSS on admission	The presence of FLAIR LA in the deep or periventricular white matter was not associated with HT
11.	Yang et al., 2018 [53]	614/ 67.4 ± 12.6	3 months	modified Van Swieten scale, ARWMC scale	Pre-intervention and within 24–36 h after IVT	within 4.5 h from symptom onset	NIHSS at baseline, mRS at 3 months	There was no significant difference in the risk of symptomatic ICH between patients with and without severe LA, regardless of having used different LA rating scales. However, severe LA was independently associated with poor functional outcomes at 3 months
12.	Liu et al., 2018 [54]	113/ 67.5 ± 10.9	90 days	Fazekas scale	On admission	within 4.5 h from symptom onset	NIHSS on admission and 24 h later, FMS at admission and at day 90	Deep WMH patients undergoing IVT had a higher risk of END (defined as an increase of ≥1 point on the motor NIHSS score or ≥2 points on the total NIHSS score 72 h after admission) and HT than deep WMH patients without IVT. Also, periventricular hyperintensities were an independent risk factor for stroke recurrence in AIS patients
13.	Chen et al., 2018 [55]	503/ 67.12 ± 12.76	3 months	WMH Volumetry	On admission and within 24 h after IVT	within 6 h from symptom onset	NIHSS on admission, mRS at 3 months	Patients with remote ICH had significantly larger WMH volumes, both periventricular and deep, than those without. WMH volume was also associated with local parenchymal hemorrhage, even after adjusting for admission NIHSS score and atrial fibrillation history. Lastly, WMH volume was associated with a 3-month poor functional outcome after adjusting for the variables of age, admission NIHSS score, and onset-to-needle time
14.	Delcourt et al., 2020 [56]	2916/ 67 ± 13	90 days	Van Swieten scale, Fazekas scale	On admission and at follow-up	within 4.5 h from symptoms onset	NIHSS at baseline, 24 h, and at day 7, mRS at 90 days	Severe LA reduced the chance of good functional outcomes, predicted any ICH and was associated with 90-day mortality
15.	Zivanovic et al., 2020 [57]	175	3 months	Presence or absence	On admission and within 24–48 h after IVT	mean of 155.6 min	NIHSS on admission, mRS at discharge and at 3 months	Independent predictors of a 3-month favorable outcome were ESUS, the absence of LA on CT, and the absence of diabetes
16.	Zhang et al., 2020 [58]	125/ 73.2 ± 8.2	3 months	Modified van Swieten scale	Pre-intervention and at 24 h after IVT and at clinical deterioration	within 4.5 h from symptoms onset	NIHSS on admission, mRS at 3 months	Severe LA was evidently associated with HT and with poor functional prognosis 3 months after IVT in elderly patients
17.	Qiu et al., 2021 [59]	175/ 67.0 ± 11.6	90 days	Fazekas scale	On admission and at 24 h after IVT	mean of 187 min from symptom onset	NIHSS on admission and at discharge, mRS at day 90	White matter hypoperfusion, reflecting the severity of LA, was independently associated with ICH after intravenous thrombolysis, but it did not increase the risk of poor prognosis
18.	Capuana et al., 2021 [60]	434/ 68.3 ± 13.5	3 months	Fazekas scale	On admission or within 24 h after IVT	mean of 180 min from symptom onset	NIHSS at baseline, mRS at 3 months	Higher Fazekas scale score was significantly associated with more severe ICH and with poor functional outcome at 90 days, but it was not an independent predictor of symptomatic ICH, mortality, or functional outcome in adjusted analyses
	**Intravenous thrombolysis and/or endovascular stroke therapy**							
1.	da Silva-Candal et al., 2020 [61]	875 (710 IVT, 87 EST, 78 both)/72.3 ± 12.2	3 months	Fazekas scale	MRI or CT study at admission and between 4th–7th day	Within 6 h from symptom onset	NIHSS at admission, every 6 h during the first day, and every 24 h during hospitalization, mRs at discharge, and at 3 months	The presence and grade of LA had a significant impact on symptomatic HT and LA degree was independently associated with poor functional outcome
2.	Hervella et al., 2021 [62]	875 (710 IVT, 87 EST, 78 both)	25 ± 13 months	Fazekas scale	CT was performed in all patients and MRI in selected patients at admission. Follow-up CT scan after reperfusion therapy was performed in all patients at 24 h, and CT at 48 h or at any time if neurological deterioration was detected; and between the 4th and 7th day	Within 4.5 h from symptom onset	NIHSS at admission, every 6 h during the first day, and every 24 h during hospitalization, mRs at discharge, and at 3 months	Greater severity of LA was independently associated with higher probability of stroke recurrence
3.	Sudre et al., 2021 [63]	1000 (199 IVT, 278 EST, 523 both)	3 months	Fazekas scale	NM	NM	NIHSS at admission, mRS at 3 months	The absence of severe Fazekas grade 3 periventricular white matter lesions was significantly associated with good outcome at 3 months

Abbreviations: AIS: acute ischemic stroke, ARWMC: Age-Related White Matter Changes, CT: Computed Tomography, FMS: Fugl-Meyer rating scale, HT: hemorrhagic transformation, END: early neurological deterioration, ENI: early neurological improvement, EST: endovascular stroke therapy, ESUS: embolic stroke of undetermined source, IAT: intra-arterial thrombolysis, ICH: intracerebral hemorrhage, IVT: intravenous thrombolysis, LA: leukoaraiosis, MRI: Magnetic Resonance Imaging, mRS: modified Rankin Scale, MT: mechanical thrombectomy, NIHSS: National Institutes of Health Stroke Scale, NM: not mentioned, OHS: Oxford Handicap Scale, WMH: white matter hyperintensity.

## Data Availability

All data discussed within this manuscript is available on PubMed.
